# Complement-Mediated Thrombotic Microangiopathy in a Patient With Antiphospholipid Syndrome and Anti-glomerular Basement Membrane Antibodies

**DOI:** 10.7759/cureus.42410

**Published:** 2023-07-24

**Authors:** Tara L Gallant, Emily Zheng, Alicia M Hobbs, Alexander J Becka, Ruth A Bertsch

**Affiliations:** 1 College of Medicine, California Northstate University College of Medicine, Elk Grove, USA; 2 Family Medicine, Kaiser Permanente, Sacramento, USA; 3 Internal Medicine, Kaiser Permanente, Sacramento, USA

**Keywords:** anti-glomerular basement membrane disease, thrombotic microangiopathy, goodpasture syndrome, antiphospholipid syndrome, acute kidney injury, complement-mediated thrombotic microangiopathy

## Abstract

Thrombotic microangiopathy (TMA) is a range of diseases characterized by thrombocytopenia, microangiopathic hemolytic anemia, and organ injury. Complement-mediated TMA is a rare, life-threatening subtype of TMA that occurs due to the uncontrolled activation of the alternative complement pathway in the absence of normal regulation, often resulting from deficiencies of various regulatory proteins. Anti-glomerular basement membrane (anti-GBM) disease, previously known as Goodpasture syndrome, is a life-threatening form of vasculitis in which immunoglobulin G autoantibodies bind to the alpha-3 chain of type IV collagen in alveolar and glomerular basement membranes. We present the case of a patient with a history of antiphospholipid syndrome who was diagnosed with complement-mediated TMA during hospital admission for elevated anti-GBM antibody titers discovered during an outpatient evaluation for elevated creatinine levels. Upon admission, treatment was started for presumed anti-GBM disease, including high-dose intravenous methylprednisolone injections and multiple plasmapheresis sessions. However, renal biopsy results showed no evidence of anti-GBM disease, but rather evidence of TMA. Subsequent laboratory studies revealed decreased complement levels, suggestive of a diagnosis of complement-mediated TMA. The patient was started on rituximab and eculizumab infusions, and she was discharged in stable condition after a 15-day hospitalization with outpatient appointments scheduled for genetic testing and further infusions. This case illustrates the importance of recognizing the key clinical and diagnostic features of complement-mediated TMA to promptly initiate appropriate therapy.

## Introduction

We present the case of a patient with a history of antiphospholipid syndrome (APS) who was diagnosed with complement-mediated thrombotic microangiopathy (TMA) during hospital admission for elevated anti-glomerular basement membrane (anti-GBM) antibody titers. Prior to her presentation, the antibodies were discovered during an outpatient evaluation for elevated creatinine levels.

TMA describes a range of diseases characterized by thrombocytopenia, microangiopathic hemolytic anemia (MAHA), and organ injury, including acute kidney injury (AKI) [1,2]. Systemic and/or intrarenal platelet aggregation causes microvascular occlusion and mechanical injury to erythrocytes, resulting in schistocyte formation [2,3]. Tissue ischemia damages organs and elevates serum levels of lactate dehydrogenase [3]. Primary causes of TMA include thrombotic thrombocytopenic purpura (TTP), hemolytic-uremic syndrome (HUS), complement-mediated TMA, drug-mediated TMA (in which a drug elicits an immune reaction or a toxic dose-related reaction), metabolism-mediated TMA, and coagulation-mediated TMA [1-3]. Secondary causes of TMA include pre-eclampsia/HELLP (hemolysis, elevated liver enzymes, and low platelets) syndrome, malignancy, systemic infection, severe hypertension, systemic lupus erythematosus, and organ transplant complications [4].

Complement-mediated TMA is a rare, life-threatening disorder characterized by thrombocytopenia, MAHA, systemic organ damage (including AKI), and often, hypertension [2,4,5]. This disorder occurs when the normal regulatory factors of the alternative complement pathway cease to function adequately, causing uncontrolled complement pathway activation. This may be caused by a deficiency of a regulatory protein called Factor H, which results in increased C3b deposition on tissues and increased formation of the C5b-9 membrane attack complex, leading to endothelial injury and platelet activation [2,4]. Eculizumab, a terminal complement inhibitor targeting C5, is considered the first-line therapy for this disorder, although evidence is limited to case series [2,4,5]. Complement-mediated TMA has been documented in conjunction with lupus nephritis both with and without concurrent APS [5].

TTP is a primary form of TMA that can be familial or acquired. TTP is a disorder defined by the deficiency of the ADAMTS13 enzyme, which is responsible for cleaving von Willebrand multimers in the plasma. This deficiency leads to systemic clumping of platelets and von Willebrand factor. Delay in recognition and treatment of the disease typically results in the progression of symptoms involving the classic pentad of thrombocytopenia, MAHA, neurologic abnormalities, renal failure, and fever [3].

Anti-GBM disease, previously known as Goodpasture syndrome, is a life-threatening form of vasculitis in which IgG autoantibodies bind to the alpha-3 chain of type IV collagen in alveolar and glomerular basement membranes [6,7]. It typically presents with hemoptysis resulting from alveolar hemorrhage in conjunction with rapidly progressive glomerulonephritis that can result in renal failure if untreated [6]. Anti-GBM disease has previously been documented in conjunction with thrombocytopenia due to immune thrombocytopenia (ITP) [8] and with TTP [1,9,10].

APS is an autoimmune condition that classically presents as pregnancy loss and/or systemic thromboses. Diagnosis is confirmed by the presence of antiphospholipid antibodies, anti-cardiolipin antibodies, and/or anti-β2-glycoprotein-I antibodies [11]. Although most clinicians regard APS as associated with large vessel thromboses, it can also result in microangiopathy [12]. Its most severe form, known as catastrophic APS, is characterized by life-threatening multisystemic organ dysfunction, usually associated with microthrombosis, and can be seen in conjunction with other TMAs [11].

## Case presentation

A 30-year-old woman presented to the emergency department (ED) at the request of her nephrologist to further evaluate an elevated IgG anti-GBM antibody titer, initiate plasmapheresis, and expedite renal biopsy. She had been referred to nephrology several weeks prior after a largely normal metabolic panel at an ED visit for a persistent, dry cough that showed elevated creatinine. Her past medical history was most notable for antiphospholipid antibody syndrome, for which she was recently started on apixaban, complex partial epilepsy, and migraines with aura. She was G4P0404 with preeclampsia in her first pregnancy and HELLP syndrome in her most recent three pregnancies. Her only symptoms upon arrival to the ED were a dry cough and foamy urine.

Upon admission, initial labs were notable for microcytic anemia with a hemoglobin of 11.1 g/dL, a creatinine of 1.83 mg/dL, a platelet count of 92,000/mm^3^, lactate dehydrogenase of 335 units/L, and a total bilirubin of 0.4 mg/dL; a computed axial tomography (CAT) scan of the chest was unremarkable. A urinalysis from five days prior to admission showed protein >500 mg/dL with no red blood cells or red blood cell casts. The patient was started on plasmapheresis and IV steroids. Apixaban was replaced by a heparin drip, and a renal biopsy was scheduled for 48 hours following her most recent apixaban dose. A tunneled plasmapheresis catheter was inserted in the right internal jugular vein, and she underwent her first plasmapheresis session with albumin on the night of admission. An ADAMTS13 titer was drawn for investigation of possible TTP the following morning due to the patient’s impaired renal function and history of coagulopathy. The first two days of hospitalization were uneventful, and the patient developed no new symptoms.

Interventional radiology performed a renal biopsy on hospital day three. The next morning, the patient developed significant thrombocytopenia with a platelet count of 40,000/mm^3^, worsening microcytic anemia with a hemoglobin of 7.6 g/dL, and pain at the biopsy site. Renal ultrasound showed a 5.3 x 2.4 x 6.9 cm hematoma. The decision was made to monitor the hematoma with serial complete blood counts. Hemoglobin and hematocrit remained stable over the following 24 hours, but platelet counts continued to decline, prompting further investigation to determine the etiology of thrombocytopenia. Due to the drop in platelets following the initiation of a heparin drip, a heparin-induced thrombocytopenia (HIT) panel was ordered, and peripheral blood smears were drawn to look for schistocytes indicative of MAHA from possible disseminated intravascular coagulation.

**Figure 1:**
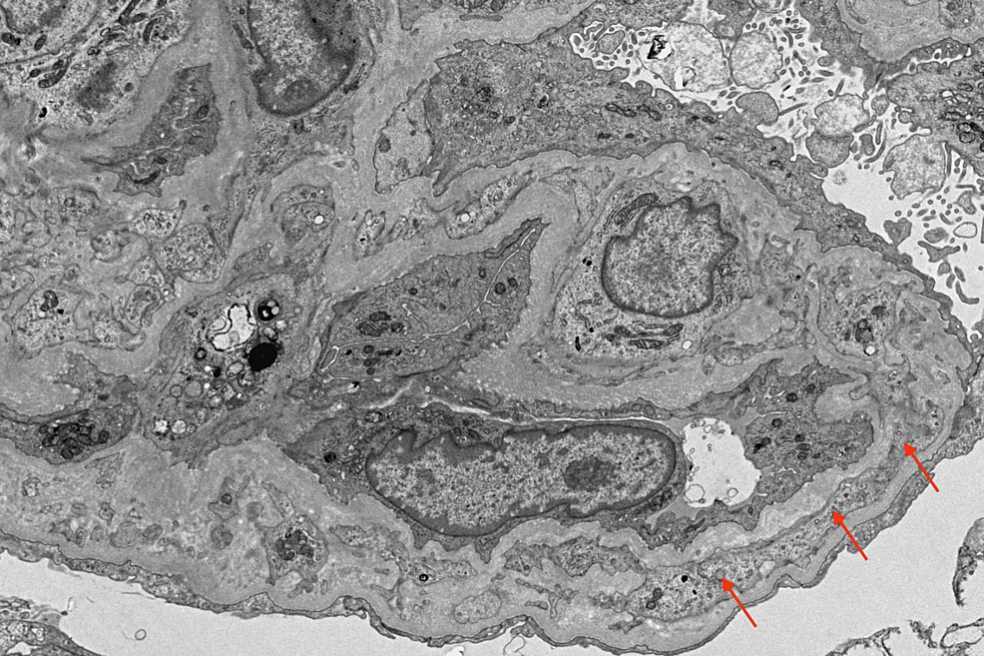
Electron microscopy demonstrates one capillary loop with duplicated glomerular basement membranes and cellular interposition between the layers (red arrows). There is diffuse endothelial cell and podocyte foot process injury.

**Figure 2:**
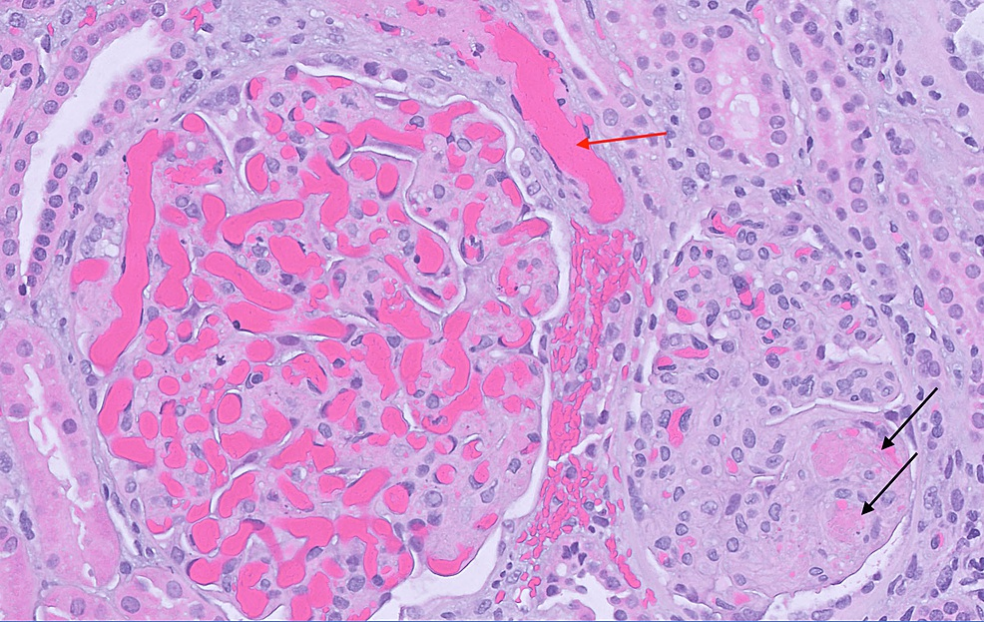
Two glomeruli with thrombotic microangiopathy. The smaller glomerulus at the right contains fibrin thrombi with nearby karyorrhexis and basement membrane duplication (black arrows). The arteriole at the top left, associated with the larger glomerulus, is filled with fibrin and red blood cells (red arrow). In the glomerulus, numerous red cells stuff the capillary lumens, but there is also endothelial cell swelling, mesangiolysis, and a few red cell fragments. H&E, original magnification 400x. H&E: hematoxylin and eosin

**Figure 3:**
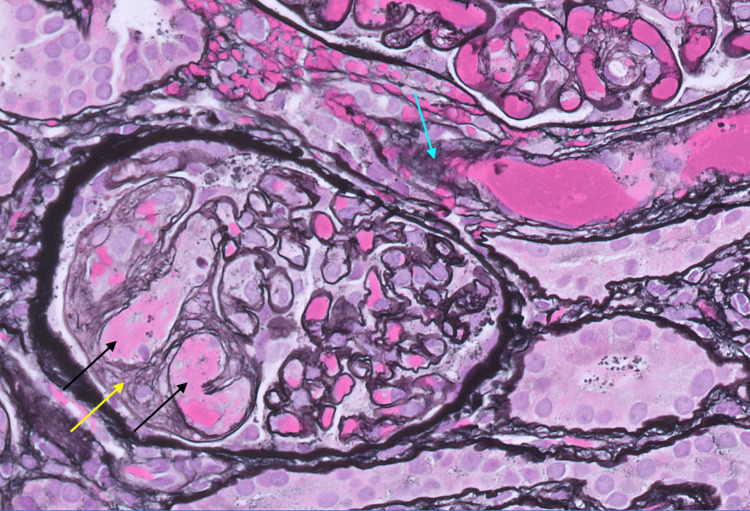
Glomeruli and arteriole with thrombotic microangiopathy. Fibrin thrombi are apparent in glomerular capillary loops at the lower left (black arrows). The glomerular basement membranes are multi-layered, consistent with chronicity (yellow arrow). The arteriole at the right contains luminal fibrin (pink) and many red blood cells (blue arrow). Jones silver stain, original magnification 630x.

Over the following two days, platelet count and hemoglobin levels improved to 110,000/mm^3^ and 8.1 g/dL, respectively, and the patient developed no new symptoms. Surprisingly, preliminary biopsy results showed no evidence of anti-GBM disease, but rather evidence of acute TMA with 50% interstitial fibrosis and tubular atrophy. Further labs were drawn to assess for complement-mediated TMA. Lab results showed decreased C3 (69 mg/dL), C4 (8.0 mg/dL), haptoglobin levels (<9 mg/dL), and elevated lactate dehydrogenase (291 units/L). A peripheral blood smear was notable for target cells and schistocytes. The HIT panel was negative, and the ADAMTS13 level was 61%, which ruled out TTP as a possible cause of TMA. Final biopsy results, shown in Figures 1-3 below, were significant for evidence of both acute and chronic TMA.

The patient was hospitalized for a total of 15 days. Once HIT was ruled out, heparin was used to bridge the patient to chronic warfarin therapy for the management of antiphospholipid antibody syndrome. Plasmapheresis continued daily. Rituximab and eculizumab were started with the hope of weaning the patient from plasmapheresis and steroids, and a slow prednisone taper was begun. Upon discharge, she had developed no new symptoms, and her creatinine was trending downward. Genetic testing to confirm the diagnosis of complement-mediated TMA was planned in the outpatient setting, and close follow-up was scheduled with her hematologist and nephrologist.

## Discussion

When the patient first presented to the ED, it was presumed that the elevated creatinine was the result of anti-GBM disease and presumptive treatment was started immediately due to the potential for alveolar hemorrhage and rapid progression to renal failure [6]. It is thought that the development of anti-GBM disease may be preceded by the production of autoantibodies triggered by an unknown stimulus such as pulmonary irritation or kidney injury, and prompt intervention is warranted for the optimization of patient outcomes [7].

The possibility of anti-GBM disease was of particular concern in this patient due to her history of APS. To the best of the authors’ knowledge, only one case report exists discussing the concurrence of these two diseases, and that the patient’s clinical course was complicated by renal failure requiring hemodialysis, alveolar hemorrhage, convulsions, and a prolonged ICU stay [13]. Hence, presumptive treatment for anti-GBM disease was begun immediately upon the patient’s arrival at the hospital in the hopes of preventing progression to alveolar hemorrhage and hemodialysis.

The renal biopsy results unexpectedly showed no evidence of anti-GBM disease. However, it has been shown that anti-GBM antibodies can exist in human sera in the absence of renal or pulmonary disease, albeit typically at lower levels than in patients with clinical evidence of anti-GBM disease [14]. Despite the absence of anti-GBM disease, it was fortuitous that treatment for anti-GBM disease was initiated as soon as the patient was admitted, as the treatment for TMA is similar [2], and the patient’s platelets improved while the team searched for the correct diagnosis.

Complement-mediated TMA is an extremely rare and likely underdiagnosed disease [4,5]. It has been previously shown to be associated with lupus nephritis and pregnancy [5], and its incidence may be increased in individuals with autoimmune diseases [4]. Although studies on complement-mediated TMA treatment are limited, previous research has shown that eculizumab therapy has the potential to induce excellent hematologic responses, as evidenced by improved platelet counts and haptoglobin levels [4]. Rituximab has also been shown to be effective in the treatment of various forms of TMA, including complement-mediated TMA [15].

## Conclusions

Our case demonstrates the importance of evidence-based medicine and effective communication among treatment teams. This case began with an ED visit for a persistent, dry cough. Instead of brushing off an incidentally elevated creatinine as unimportant, the emergency physician ordered a follow-up creatinine, which led to the patient’s referral to nephrology, and then to her hospitalization. Her team diligently considered a wide range of potential causes for elevated creatinine levels, a critical approach for patients with a coagulopathy history. Complement-mediated TMA is infrequent, and it is often not prioritized in the initial assessment of patients with proteinuria and elevated creatinine. Nevertheless, timely intervention can significantly enhance patient outcomes, and any delay in diagnosis and treatment could have adversely affected this patient’s prognosis.

Our patient, diagnosed with APS, was initially hospitalized due to suspected anti-GBM disease. Plasmapheresis and high-dose corticosteroids were administered as treatment. However, a kidney biopsy excluded anti-GBM disease and instead revealed a diagnosis of TMA, specifically complement-mediated TMA. Subsequently, the patient’s treatment plan included the initiation of rituximab and eculizumab infusions, with the intention to continue them indefinitely. Although rituximab and eculizumab have demonstrated effectiveness in treating both complement-mediated TMA and other forms of TMA, the available data primarily consists of case series. Previous case series have shown significant improvement in renal function, anemia, and thrombocytopenia with eculizumab. However, further research in this field is needed to aid healthcare providers in diagnosing patients and enhancing treatment guidelines.
